# Comparison of cervical cancer screening by self-sampling papillomavirus test versus pap-smear in underprivileged women in France

**DOI:** 10.1186/s12905-021-01356-8

**Published:** 2021-05-26

**Authors:** Laura Reques, Camille Rolland, Anne Lallemand, Najat Lahmidi, Ezequiel Aranda-Fernández, Antonio Lazzarino, Julie Bottero, Françoise Hamers, Christine Bergeron, Ken Haguenoer, Guy Launoy, Niklas Luhmann

**Affiliations:** 1 Médecins du Monde, 62 rue Marcadet, 75018 Paris, France; 2 EPISTATA – Agency for Clinical Research and Medical Statistics, London, UK; 3 Unité de Maladies Infectieuses et Tropicales, Groupe Hospitalo-Universitaire Paris Seine St-Denis, AP-HP, Hôpital Jean Verdier, Bondy, France; 4 Santé Publique France (National Public Health Agency), Saint Maurice cedex, France; 5 Laboratoire CERBA, Saint-Ouen-l’Aumône, France; 6 U1153, INSERM, Paris, France; 7 Cancer Screening Department, CHRU de Tours, 37000 Tours, France; 8 Centre François Baclesse, INSERM, Avenue du Général Harris, 14076 Caen, France

**Keywords:** SC-HPV, Papillomavirus, Cervical cancer, Screening, Vulnerability

## Abstract

**Background:**

The purpose of this study was to compare cervical cancer screening by pap smear (PS) versus preliminary HPV testing based on self-collected samples (SC-HPV).

**Methods:**

Interventional study among underprivileged women from 25 to 65 years old in four French cities. The control group (CG) was referred for a PS. The experimental group (EG) conducted a SC-HPV test followed by a PS in case of positivity. Differences on screening completion and cytological abnormalities were analysed by logistic and Cox regression.

**Results:**

383 women were assigned to the EG and 304 to the CG. The screening completion proportion was 39.5% in the CG compared to 71.3% in the EG (HR = 2.48 (CI 95% [1.99–3.08]; *p* < 0.001). The proportion of cytological abnormalities was 2.0% in the CG and 2.3% in the EG (OR = 1.20 (CI 95% [0.42–3.40]; p = 0.7). The proportion of participants lost to follow-up was 60.5% in the CG and 63.2% in the EG HPV positive (*p* = 0.18).

**Conclusion:**

Providing an SC-HPV-test increased the participation of underprivileged women in CCS. Nevertheless, the significant number of lost to follow-up in both groups can undermine the initial benefits of the strategy for HPV positive women.

Registration: Clinicaltrials.gov: NCT03118258.

## Background

Human papillomavirus (HPV) is the most common sexually transmitted infection [1]. In most cases, it is asymptomatic and can regress spontaneously. Nevertheless, an infection by a high-risk genotype (HR-HPV) can persist and might result in precancerous lesions that might turn into cervical cancer after 5 to 20 years [1–4].

Cervical cancer is the most frequently diagnosed type of cancer among women in 28 countries and the leading cause of cancer-related mortality in 42 countries, most of which are located in Sub-Saharan Africa and South-East Asia [5].

In France, cervical cancer affects nearly 3000 women and causes around 1100 deaths every year [6]. Currently, pap smear (PS) is the primary cervical cancer screening (CCS) test used to detect precancerous lesions and early-stage cervical cancer [3, 7]. European guidelines set the acceptable level of coverage at 70% and the desired level at 85% [8]. National three-year coverage rate of PS screening is estimated to be insufficient (60%) [9].

The French national cervical cancer screening program relies directly on health professionals providing gynaecological services to the women concerned, mainly gynaecologists, general practitioners and midwives. The strategy is accompanied by communication campaigns and individual invitations for target population (women between 25 and 65 years old). Cytology exams and HPV tests are covered 100% for insured women. This strategy ostracises women without health coverage or experimenting health care access difficulties. Actually, it is estimated that women with limited access to the healthcare system, women of low socio-economic status, and women living in economically depressed areas are less likely to have access to CCS [10, 11].

Data from Médecins du Monde (MdM) indicated that nearly 67% of women between the ages of 25 and 65 attending to MdM programmes in France had never performed a PS [12]. This finding has been supported by other studies [13]. In addition, these underprivileged women often come from countries with the highest cervical cancer incidence and mortality rates [5]. The concept “underprivileged” joins the notions of low social and economic status, including low income, low educational level, migration status, unemployment, shelter conditions, minorities, absence of social insurance or marginalisation.

HPV testing has been shown to be more sensitive than the PS exam [14–16]. Self-collected samples for HPV testing (SC-HPV) have been found to be as sensitive as clinically-collected samples [17]. Several international studies described the SC-HPV technique as easier, less painful, less bothersome and faster than a conventional PS [18, 19]. Based on these findings, several Western countries have recently incorporated HPV testing into their CCS strategy. In 2015, the European Commission recommended to include a two-phase HPV testing strategy in an organised screening programme [8].

The purpose of this study was to compare the proportion of screening test completion and the proportion of cytological abnormalities detected using two different strategies within underprivileged women in France. These strategies included an individual preventive medical consultation followed by either a referral to a partner facility for a PS or preliminary SC-HPV testing.

## Methods

### Study type

Interventional, multicentre, comparative, and randomised research focused on four types of programmes: CASO (Reception, Healthcare, and Orientation Centres), CAOA (Reception, Orientation, and Support Centres), Squats/Slums, and Sex Worker programmes (SWP) in four cities (Lyon, Bordeaux, Rouen, and Paris).

MdM's CASO/CAOA are structures conceived to facilitate access to healthcare rights and prevention for people experimenting socioeconomic difficulties. They have permanent and voluntary staff, including doctors, nurses, psychologists, social workers and community agents. Users are mainly newly arrived migrants, but also other profiles with social factors of vulnerability (like homeless, unemployed and drug users). People come spontaneously to the centres through multiple community communication channels.

The other programs include outreach actions, particularly in vulnerable settlements (squats and slums) and in sex workers place of practice. In the slums, MdM offers on-site medical and prevention consultations accompanied by referrals to other healthcare structures and sensitization about contraception, family planning and prenatal care. Programs with sex workers address the topics of sexually transmitted infections, hepatitis, HIV, unwanted pregnancies, violence, psychological suffering and advocacy.

Programmes participating in the study were selected to have representative profiles and locations according to the flux of women in the targeted age range and to the presence of sexual and reproductive activities in the programmes’ current line-up of services.

The study was conducted from March 2017 to December 2018.

### Inclusion criteria

All women between the ages of 25 and 65 who had not had a PS in the past three years were included in the study. Exclusion criteria included study participation refusal, having a history of a complete hysterectomy or the absence of sexual intercourse in the past.

### Participant screening process

Eligible women were offered an individual sexual and reproductive health (SRH) consultation which included an adapted and individual consultation informing about HPV infections, cervical cancer, the importance of gynaecological care and screening strategies. An interpreter or healthcare mediator was also available. At the end of the consultation, women were separated into two groups: (1) The “PS” control group (CG) (in which study participants were referred towards partner associations and institutions to have a PS); (2) The “SC-HPV” experimental group (EG) (in which HPV was tested using a self-collected sample, followed by a referral to partner associations for a PS if the HPV results were positive or non-interpretable/missing). EG patients were provided with a SC-HPV kit and given the choice of either collecting the sample on site in a private room or at home (in the case of menstruation or if the patient declined to collect the sample on site). Patients were given their HPV results two weeks later, and they were scheduled for a follow-up appointment. If results were positive, patients were directed to partner centres to get a PS. Women whose HPV tests were positive received a reminder phone-call. Patients assigned to CG benefited systematically from a HPV test when cytological abnormalities were detected in the PS.

Women who had not completed an HPV test or PS four months after their appointment were considered lost to follow-up.

### Sample size

Sample size calculation was based on risk a = 5% and power of 80%, a proportion of CCS completion of 80% in the EG and 40% in the CG, and a proportion of cytological abnormalities of 2.5% in the CG and 4% in the EG. The number of participants was estimated at 1258 to answer to both objectives.

### Randomisation

Participants in each programme were randomly assigned over one-month periods. Each month in which participants were offered an SC-HPV was followed by a period in which participants were not offered this option.

### Biological analyses

The test selected for the study was the ABBOTT Real Time High Risk HPV test (real-time PCR test detecting 14 HPV genotypes: 16, 18, 31, 33, 35, 39, 45, 51, 52, 56, 58, 59, 66, and 68). All partner facilities conducted liquid-based cytologies.

### Data collection

Participants’ medical and social data were collected through a questionnaire administered by trained professionals. These data were entered into a secure “electronic patient record” tool. MdM’s partner structures submitted the results of the PS exams via confidential mail.

### Evaluation criteria

Screening completion rate was defined as the proportion of women who have a PS done in the CG and the proportion of women who had a negative HR-HPV test or had a PS done if the HR-HPV test was positive in the EG.

As cytological abnormalities we considered ASC-US, LSIL and HSIL.

### Statistical analysis

The descriptive analysis was conducted based on the demographic, socio-economic, and clinical variables in each procedure group. Comparability of the groups was evaluated using Chi-2 tests, ANOVA tests for categorical variables whereas t-Student tests for continuous variables. The comparison between the rate of screening completion and the number of cytological lesions was conducted using logistic and Cox regressions. All analyses were repeated with another sample that only included data from CASO/CAOA programmes. Statistical analysis was done using the software Stata 15 [20].

### Ethical considerations

This research project was approved by the Île de France IV Institutional Review Board (IRB). The study was conducted in compliance with the ethical principles of the Helsinki Declaration. All participants were informed of the study’s objectives and design, and their participation was voluntary after providing verbal informed consent. The protocol was registered in clinicaltrials.gov.

## Results

**Fig. 1:**
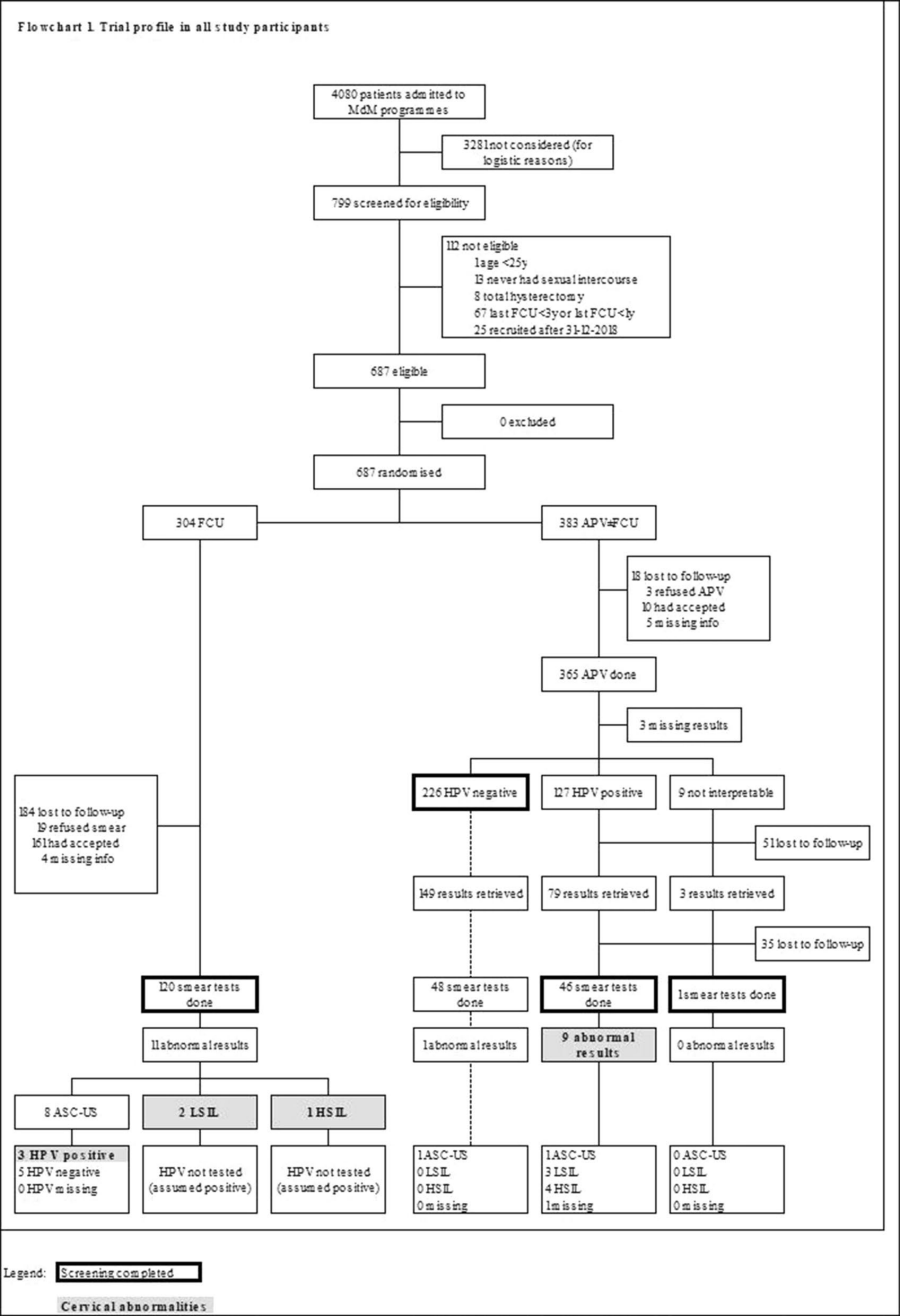
Study flowchart

The study flowchart is depicted in Fig. 1. Out of the 799 participants in the study who received a gynaecological consultation, 112 (15.3%) were not eligible for the intervention (67 were up to date with their CCS, 13 had never had sexual relations, and 8 had had a complete hysterectomy). The final population was 687 women, 304 of which were assigned to the CG (PS) and 383 to the EG (HPV self-sample). The EG was slightly larger because of a higher participation rate among participants from the SWP in Paris during the months when SC-HPV was offered.

In the CG, out of the 304 women who were referred for a PS, 120 (39.5%) followed through with the exam and 184 (60.5%) were lost to follow up. Out of the 120 PS conducted, 11 cytological abnormalities were detected: 8 ASC-US (3 of which were caused by an HPV infection), 2 LSIL, and 1 HSIL.

In the EG, out of the 383 women, 365 (95.3%) submitted SC-HPV, 15 (3.9%) were lost to follow-up, and three (0.8%) declined to submit a self-sample. Out of the HPV tests that were completed, 226 were negative, 127 were positive, 9 could not be interpreted, and 3 were missing. 34.4% of the HPV-tests done were positive. Out of the 136 women who tested positive for HPV or whose test could not be interpreted, 47 (34.6%) had a PS and 9 cytological abnormalities were detected (1 ASC-US, 3 LSIL, and 5 HSIL).

In the CG, 184 women were lost to follow-up after they were given a referral for a PS (60.5%). In the EG, 18 women were lost to follow-up before they could submit an SC-HPV (4.7%). In addition, out of the 136 women whose HPV results were positive or could not be interpreted, 35 were lost to follow-up after they were referred for a PS (63.2% of women with an indication to have a PS).

Table 1 presents a description of the characteristics of the participants by group: 54.7% had completed up to either primary or secondary school, 62.5% had been in France for under a year, 67.7% did not have a reported employment, 73.4% were undocumented, and 68.3% did not have health insurance. 40% needed interpreting services. There were no statistically significant differences between the main covariables in the two groups.

**Table 1:** Characteristics of study participants by procedure group

Variable and category	Total		Procedure group				*p* value
			Control		Experimental		
	N = 687		N = 304		N = 383		
Age	41.0	(SD 10.1)	39.7	(SD 9.9)	42.0	(SD 10.2)	0.004
*Educational level (%)*							0.81
Primary school or less	27.0	(183/678)	27.7	(83/300)	26.5	(100/378)	
Secondary school	54.7	(371/678)	53.3	(160/300)	55.8	(211/378)	
University	18.3	(124/678)	19.0	(57/300)	17.7	(67/378)	
*Time spent in France (%)*							0.008
< 3 months	32.5	(217/668)	33.4	(101/302)	31.7	(116/366)	
3–12 months	30.5	(204/668)	35.4	(107/302)	26.5	(97/366)	
> 12 months	37.0	(247/668)	31.1	(94/302)	41.8	(153/366)	
Employment (%)	27.4	(180/658)	19.1	(57/298)	34.2	(123/360)	< 0.001
*Migration status (%)*							0.60
Documented	16.9	(113/669)	15.0	(45/301)	18.5	(68/368)	
Undocumented	73.4	(491/669)	74.4	(224/301)	72.6	(267/368)	
In the process of becoming legal	8.4	(56/669)	9.3	(28/301)	7.6	(28/368)	
Unknown	1.3	(9/669)	1.3	(4/301)	1.4	(5/368)	
Need for an interpreter (%)	40.3	(273/677)	33.6	(100/298)	45.6	(173/379)	0.001
*Health coverage (%)*							0.17
None	68.3	(457/669)	72.7	(218/300)	64.8	(239/369)	
PUMa (French universal healthcare regime)	4.6	(31/669)	4.7	(14/300)	4.6	(17/369)	
Top-off insurance	6.7	(45/669)	6.3	(19/300)	7.0	(26/369)	
AME (healthcare social worker)	19.6	(131/669)	16.0	(48/300)	22.5	(83/369)	
Other	0.7	(5/669)	0.3	(1/300)	1.1	(4/369)	
Number of dependent children	1.1	(SD 1.3)	1.1	(SD 1.3)	1.1	(SD 1.2)	1.00
Screening test completion (%)	57.2	(393/687)	39.5	(120/304)	71.3	(273/383)	< 0.001
Cytological abnormalities detected (%)	2.2	(15/687)	2.0	(6/304)	2.3	(9/383)	0.74

With respect to the proportion of screening completion, the participants in the EG were approximately twice as likely to have access to the test as the CG (39.5% compared to 71.3%, *p* < 0.001), with a risk ratio (RR) of 1.80: CI 95% [1.55–2.10]; *p* < 0.001.

**Fig. 2:**
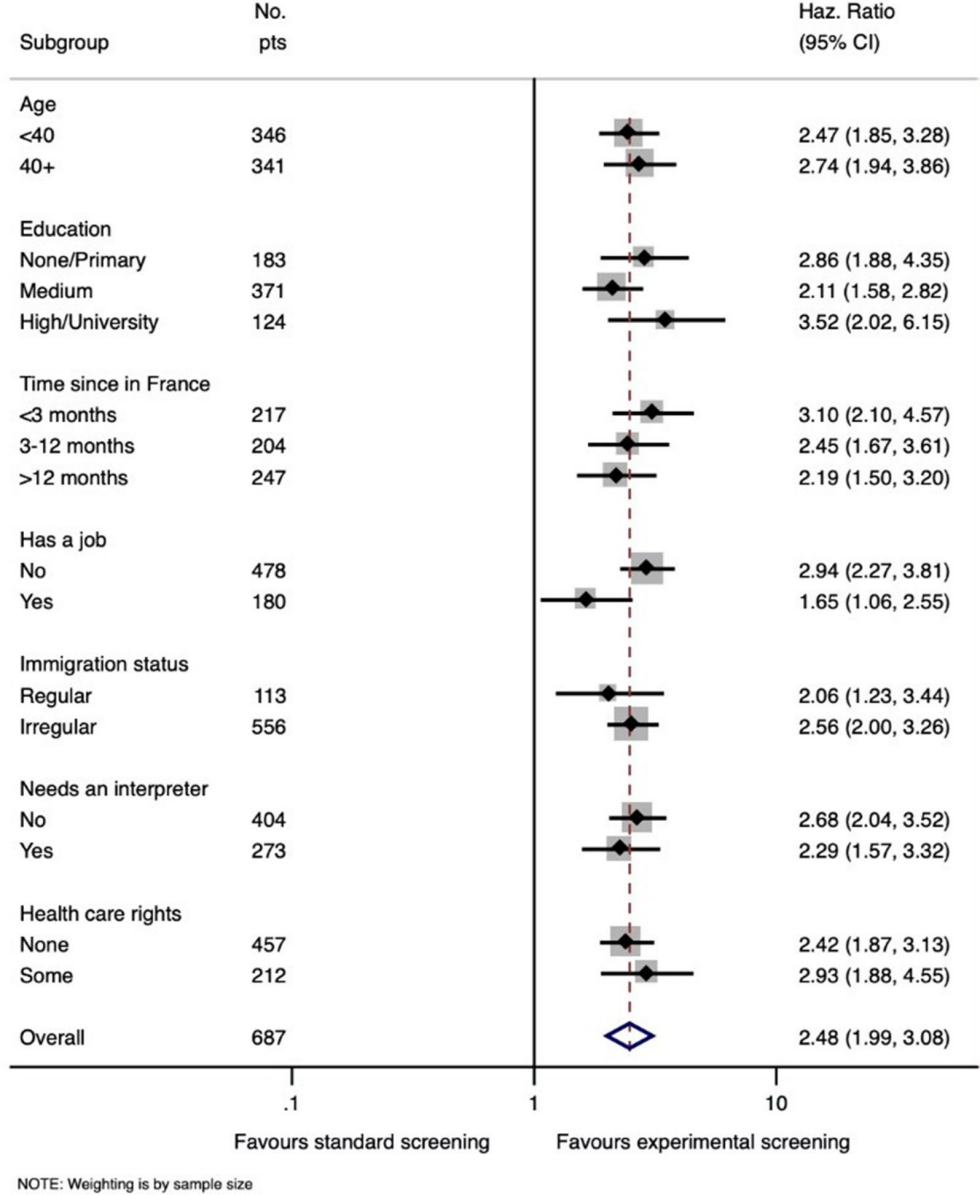
Sub-group analysis using a forest plot representing the cervical cancer screening completion rate

Figure 2 shows a forest plot including an analysis with respect to the screening test completion. Among the women who completed the screening tests, the process took an average of 18.6 days (SD: 34.5) for the CG and 9.5 days (SD: 23.8) for the EG (*p* < 0.001).

The hazard ratio (HR) for the screening test completion rate for the EG compared to the CG was 2.48 (CI 95% [1.99–3.08]; *p* < 0.001). The analysis of the sub-groups shows that the effect was not modified by the study’s other covariables.

**Fig. 3:**
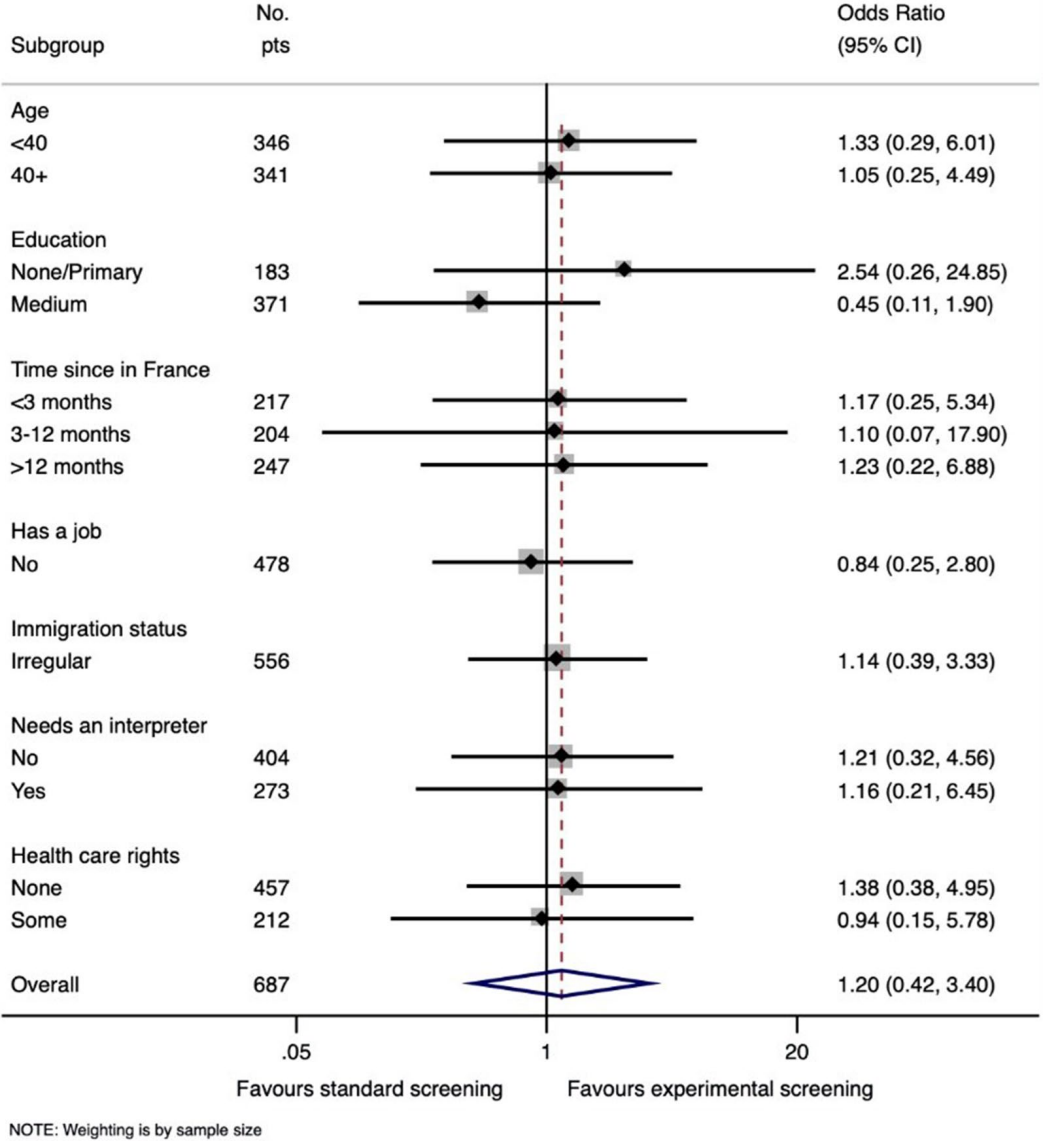
Sub-group analysis using a forest plot representing the detection of cytological cervical abnormalities

Figure 3 shows a forest plot that includes an analysis of the overall data set and that of each sub-group with respect to the number of cytological abnormalities detected. In the CG, 2.0% of women had cytological abnormalities associated with an HPV infection, and 2.3% in the EG, with an OR of 1.20 (0.42–3.40), *p* = 0.7. The power of this difference in proportions was weak (0.056). The effect was not modified by other study’s covariables.

All analyses were also conducted using a sample that only included participants from programmes with more consistent results (CASO/CAOA). Both groups were well balanced and their results do not differ from the results of the entire sample in terms of screening performance, number of participants lost to follow-up, screening test completion, and cytological abnormalities detected (Appendices 1, 2, 3, 4).

## Discussion

The option of using a SC-HPV is an effective alternative for improving screening access and coverage among underprivileged women. Nevertheless, the number of required visits makes it difficult to complete the screening process, which limits the strategy’s benefit.

The profile of the women who participated in the study varies widely in terms of age, origins, and other socio-demographic aspects. This profile corresponds to the characteristics of the women who participate in MdM’s programmes, who are mainly immigrants, homeless, sex workers, and drug users. These groups have limited access to housing and healthcare. In many cases, they are also not legally employed and undocumented [12]. Socio-cultural factors, such as the individual’s country of origin, language, religion, marital and inter-familial relationships, also influence access to CCS, as it has been shown in the literature [21–27].

SC-HPV is an effective time and resource-saving strategy as it helps engaging women at preliminary stages of the screening process. This strategy has been described to be especially popular among women living in vulnerable situations [14, 28–30] and just as sensitive and slightly less specific than testing for HPV with a cervical sample taken by a physician [17, 31, 32]. In our study, the technique also had a high level of acceptability, as only 0.8% of women declined to submit a sample. In addition, the percentage of tests found to be invalid due to their transportation conditions, the quantity or quality of the material, or sampling errors was very low (< 1%). This also shows the excellent performance of these tests in real conditions and among the women participating in the study.

In July 2019, the French National Authority for Health recommended that HPV testing be included in France’s CCS strategy for women over 30 who had not been screened or who had received insufficient screening [33]. Our results show the ability of this technique to provide underprivileged women with access to CCS.

Compared to national figures, which estimate that 15–20% of women between the ages of 25 and 65 have this type of virus [10], this study showed potentially higher rates for carcinogenic HPV infection (35%). A similar finding is reported in the scientific literature, which found that higher proportion of HPV infection among sex works [34–36] and immigrant communities. The prevalence of the virus in the women’s entourage, exposure to high-risk practises, and a decreased use of prevention methods could explain these findings.

To complete the entire screening pathway, women had to attend three appointments (prevention consultation, an appointment at the partner centre for a PS exam, and an appointment to receive the results). This number might have been even higher if women needed to receive additional tests, undergo a colposcopy, or receive treatment for their lesions. In the EG, the two-stage screening strategy (HPV screening as the primary exam, delivery of the HPV test results, and PS), shortened the screening process for women whose HPV results were negative but added an extra visit in the event of positive HPV results. As a result, one of the most notable findings of our study was the high number of women lost to follow-up. It should be noted that this took place in both study groups, regardless of whether or not the women knew their HPV results. Results delivery delays (10 days for the HPV tests and a few weeks for the PS exams) added another barrier. The high number of participants who were lost to follow-up underscores how difficult it is to treat these women and could undermine the benefits gained by increasing primary screening coverage by using SC-HPV.

On the other hand, it is clearly beneficial for women to know that they tested negative for HR-HPV. Not only is it a source of relief, but it also means they can complete their screening process more quickly. Moreover, the high level of acceptability and logistical simplicity of this technique could also increase long-term participation in screening programmes.

In this sense, the benefits of HPV test approach are evident and the endpoint “participation” is absolutely clear, as the self-collected HPV test offer much more protection.

Several studies have shown that the long duration of the screening process represents a barrier to completing it within underprivileged women [36, 37]. In addition, a lack of knowledge and awareness, personal reticence, lack of time, absence of family support, need for marital consent, language barriers, travel issues, limited access to healthcare facilities, anxiety about receiving a cancer diagnosis and its consequences have also been mentioned as frequent barriers to screening access [35, 38, 39]. However, reducing the number of appointments, providing rapid testing options, points of care and improving the accessibility of healthcare facilities improves screening completion rates and patient treatment [38, 39].

Even though some of the barriers are directly related to the access to healthcare facilities, also essential for completing the screening programmes are socio-cultural determinants, understanding of the message plus the individual’s own risk perception. This reality highlights the importance of counselling and interpreting services.

### Limitations

This study has a certain number of limitations. First, monthly randomisation was selected in collaboration with teams on the field together with the scientific committee. Choosing to alternate between one-month periods was easier to follow and helped to limit bias related to seasonal variations. In the SWP in Paris, where the sex worker community is highly cohesive, offering SC-HPV during specific periods encouraged the women to visit the healthcare structures. Nevertheless, this pattern does demonstrate the benefit of this screening strategy for this population.

In addition, there were low participation rates in the Squat programmes and SWP in Rouen. This fact can be due to the different approach of the activities (CASO/CAOA are fix and stables programs with a constant influx of patients and potential candidates while Squat and SWP are based on an outreach approach, which makes mobilisation more difficult).

To limit these potential sources of bias, we decided to conduct a supplementary analysis that only included data from the CASO/CAOA (77% of the sample), obtaining very similar results to the full dataset.

The study was underpowered for the outcome “proportion of cytological abnormalities”, as the study had to stop before sample size completion due to budget constraints.

In addition, it would have been better to measure the completion rate based on the number of retrieved results, which would have ensured that the women knew their status. Unfortunately, this information was available for the results of the HPV tests (which were conducted and collected within MdM facilities), but not for the results of the PS exams (which were conducted and collected in a variety of partner facilities).

Finally, it would be beneficial to offer a holistic SRH strategy to these populations, including vaccinations and testing for other STIs, such as HIV and hepatitis.

## Conclusions

Our study is the first to provide information about different CCS strategies for underprivileged women in France.

SC-HPV strategy presented a higher proportion of screening test completion and cytological abnormalities detection (with difference of proportions non-statistically significant for the second conclusion).

Providing participants with a SC-HPV kit improved the participation of underprivileged women in CCS. Nevertheless, the significant number of lost to follow-up in both groups can undermine the initial benefits of the strategy for HPV positive women.

It is important to better understand the barriers to screening encountered within this population and thus to design more adapted, differentiated, and appropriate strategy to reduce inequalities.

## Data Availability

Data supporting the findings of this study are available within the article and its supplementary materials. Additional data are available from the corresponding author, upon reasonable request.

## Appendix 1

See Table 2.

**Table 2:** Characteristics of study participants based on procedure group: CASO and CAOA programmes only

Variable and category	Total		Procedure group				*p* value
			Control		Experimental		
	N = 497		N = 250		N = 247		
Age	38.7	(SD 9.9)	38.6	(SD 9.8)	38.8	(SD 10.0)	0.83
*Educational level (%)*							0.81
Primary school or less	29.4	(144/490)	29.3	(72/246)	29.5	(72/244)	
Secondary school	47.1	(231/490)	48.4	(119/246)	45.9	(112/244)	
University	23.5	(115/490)	22.4	(55/246)	24.6	(60/244)	
*Time spent in France (%)*							0.20
< 3 months	43.2	(210/486)	39.9	(99/248)	46.6	(111/238)	
3–12 months	37.0	(180/486)	40.7	(101/248)	33.2	(79/238)	
> 12 months	19.8	(96/486)	19.4	(48/248)	20.2	(48/238)	
Employment (%)	13.6	(66/484)	12.1	(30/247)	15.2	(36/237)	0.33
*Migration status (%)*							0.89
Documented	16.6	(81/487)	15.8	(39/247)	17.5	(42/240)	
Undocumented	71.7	(349/487)	72.1	(178/247)	71.2	(171/240)	
In the process of becoming legal	10.3	(50/487)	10.9	(27/247)	9.6	(23/240)	
Unknown	1.4	(7/487)	1.2	(3/247)	1.7	(4/240)	
Need for an interpreter (%)	18.3	(89/487)	20.1	(49/244)	16.5	(40/243)	0.30
*Health coverage (%)*							0.20
None	83.8	(409/488)	82.6	(204/247)	85.1	(205/241)	
PUMa (French universal healthcare regime)	3.5	(17/488)	4.0	(10/247)	2.9	(7/241)	
Top-off insurance	4.7	(23/488)	3.6	(9/247)	5.8	(14/241)	
AME (healthcare social worker)	7.2	(35/488)	9.3	(23/247)	5.0	(12/241)	
Other	0.8	(4/488)	0.4	(1/247)	1.2	(3/241)	
Number of dependent children	1.2	(SD 1.3)	1.1	(SD 1.3)	1.3	(SD 1.3)	0.31
Screening test completion (%)	57.3	(285/497)	38.8	(97/250)	76.1	(188/247)	< 0.001
Cytological abnormalities detected (%)	1.8	(9/497)	1.6	(4/250)	2.0	(5/247)	0.72

The estimates are expressed as mean with the standard deviation (SD) in parentheses or proportions with the absolute numbers between parentheses. The *p* values for the categorical and binary variables were calculated using Pearson’s chi-squared test. *P* values for the continuous variables were calculated using the two-sample t-test

## Abbreviations

ASC-US: Atypical squamous cells of uncertain significance
CAOA: Centre d’Accueil, Orientation et Accompagnement
CASO: Centre d’Accueil, Soins et Orientation
CG: Control group
CI: Confidence interval
CS: Control strategy
EG: Experimental group
ES: Experimental strategy
IRB: Institutional review board
HIV: Human immunodeficiency virus
HPV: Human papillomavirus
HR: Hazard ratio
HR-HPV: High-risk human papillomavirus
HSIL: High-grade squamous intraepithelial lesion
LSIL: Low-grade squamous intraepithelial lesion
MdM: Médécins du Monde
OR: Odds ratio
PS: Pap smear
RR: Risk ratio
SC-HPV: Self-collected human papillomavirus sample
SRH: Sexual and reproductive health
STI: Sexually transmitted infections
SWP: Sex worker’s programs
